# The challenges of coeliac disease at work: A contestation of the politics of inclusion

**DOI:** 10.1111/1467-9566.13826

**Published:** 2024-07-29

**Authors:** Anne Steinhoff, Rebecca Warren, David Carter, Jason Glynos

**Affiliations:** ^1^ Essex Business School University of Essex Colchester UK; ^2^ Canberra Business School University of Canberra Canberra Australian Capital Territory Australia; ^3^ Department of Government University of Essex Colchester UK

**Keywords:** coeliac disease, exclusion, inclusion, logics of equivalence, othering

## Abstract

By focusing on the experiences of employees living with coeliac disease as evidenced in UK employment tribunal cases, this paper interrogates the way practices of exclusion are performed in legal and organisational contexts that purport to promote values of inclusion. In paying attention to how differences are constructed and negotiated, the paper unpacks the way organisational practices mobilise an array of workplace mechanisms to produce complex dynamics of exclusion. Applying Laclau and Mouffe’s logics of equivalence and difference, we show how questionable impulses and practices emerge in a workplace environment characterised by unclarity and vagueness. One impulse, for example, involves privatising and individualising the condition of employees with coeliac disease, giving rise to patronising and stigmatising attitudes that can turn them into victims. However, we also identify workplace mechanisms countering these tendencies, which can underpin forms of collective support in the struggle for recognition. Our study thus contributes to the body of sociological literature that pays attention to health‐related workplace injustices by challenging the purported promotion of health‐based inclusion through a focus on tribunal cases, leading to suggestions for further research into the way medical conditions are theorised and ‘lived’ at work.

## INTRODUCTION

Workplaces are powerful social institutions in which routinised norms, behaviours and practices toward health can lead to workplace inequality and injustice. These patterns of activity can have troubling, sometimes severe, physical and mental consequences for employees who are often unable to counteract these forces on their own (Dew & Taupo, 2009; Remnant et al., 2023). When exploring the material and social conditions of organisational life (Dale & Latham, 2015), the physical body can be understood as a place where the enactment of such injustice and inequality takes place, typically through exclusion (van Amsterdam et al., 2023). Indeed, important work illustrates how exclusions tend to be enacted by forcing subjects into categories structured around dualisms, such as suitable‐unsuitable, fit‐unfit and well‐unwell (Ciuk et al., 2022), which enable norms of (embodied) Othering to emerge (Mik‐Meyer, 2016).

In this article, we contribute to this literature by showing how material and social differences associated with coeliac disease can be discursively mobilised to enact exclusions. Counter‐intuitively, organisational mechanisms responsible for creating particular exclusions often coexist with efforts to promote inclusion. We show how these mechanisms operate against a broader medical, social and legislative backdrop, suggesting that such exclusions and the mechanisms that underpin them, are more deeply rooted than we might expect and thus not resolvable by giving organisations more time to address them.

To study the exclusionary impacts sometimes accompanying a more general ‘politics of inclusion’, we turn to UK employment tribunal cases involving coeliac disease. This is because such cases offer detailed accounts of how peoples lived experiences in the workplace bump up against (contestable) organisational norms. We thus treat the UK employment tribunal as a space of public contestation, offering a unique window into the dynamics of exclusion and inclusion in workplaces. To elucidate these dynamics, we draw upon Laclau and Mouffe’s (2001) conceptualisation of ‘logics of equivalence and difference’, which have previously been used by scholars to shed light on a wide range of practices, including health policy practices (Speed & Mannion, 2020).

Our contribution is two‐fold. First, we offer a political discourse‐based perspective within which to explore the debates surrounding health‐related exclusion and injustice (Gunnarsson Payne & Korolczuk, 2016; Speed & Mannion, 2020). As a study of the manifestation of exclusion in workplace practices, we build on and contribute to, literature that adopts perspectives anchored in the idea of Othering (Remnant et al., 2023). We also draw attention to employment tribunals as relevant spaces in which scholars can study the public contestation of organisational norms and gain insight into workplace practices. Second, we offer a way to understand the complex processes that produce workplace challenges and impasses (Dew & Taupo, 2009; Remnant et al., 2023) in relation to coeliac disease (Veen et al., 2013), as revealed in the manner UK employment tribunals negotiate the tensions between organisational practices and the legal parameters of discrimination.

The paper is structured as follows. First, to understand why coeliac disease can constitute a challenge in the workplace, we outline the wider context of the disease. We then review current debates regarding health‐based exclusionary practices in the workplace and sketch out how our theoretical perspective helps shed light on these processes. The subsequent section outlines our methodology, after which we conduct our theoretically‐informed analysis of extracts from employment tribunal decisions. Our analysis identifies key mechanisms that appear to structure the workplace dynamics of exclusion and inclusion centred on coeliac disease. Finally, we discuss our findings and analysis against the background of existing literature, concluding with reflections on how our contributions might prompt future research.

## COELIAC DISEASE IN CONTEXT

To better appreciate the significance that coeliac disease plays in workplaces, we sketch out its broader medical, legal and social context. Coeliac disease is the only autoimmune disorder where the medical profession has identified and isolated its trigger: the protein gluten (Gregory, 2005). In coeliac disease, gluten causes the body to attack digestive tissues and neurological systems, producing potentially serious health complications. There is currently no cure or medications for coeliac disease, so treatment plans seek to eliminate gluten to enable tissue and neurological recovery. However, the smallest trace of gluten (a breadcrumb) can trigger symptoms and tissue damage (Coeliac UK, 2021).

While coeliac disease affects 1% of the world population (Bozorg et al., 2022), a feature of the disease is that it presents differently for different people. Some individuals live with asymptomatic coeliac disease (Coeliac UK, 2021), but for those with symptomatic coeliac disease, symptoms can return and remain for an extended period if an autoimmune reaction is triggered.

Beyond these medical challenges, there is confusion in the UK law concerning the status of coeliac disease in employment contexts. Coeliac UK (2021), the largest UK‐based charity supporting those diagnosed with coeliac disease, stresses that maintaining a gluten‐free diet can be challenging as there is no clear legal duty imposed on employers, restaurants or hospitals to cater for coeliac disease. While the Office for Disability Issues (ODI) (2011, p. 8) suggests that the Equality Act 2010 (EA, 2010, p. 14) refers to ‘auto‐immune conditions’, the Department of Health and Social Care (DHSC) (2018, p. 14) states that ‘coeliac disease is not defined as a disability under the EA 2010’ even though it acknowledges that it is ‘a long‐term condition’. This ambiguity is also present in employment tribunals which demonstrate inconsistent decision‐making around its status in their judgments.

It is notable that Coeliac UK does not mention the word disability on their website nor does the charity provide any information on workplace discrimination. Indeed, the scepticism expressed by advocates toward identifying coeliac‐afflicted individuals as disabled furnishes a reason for why one should hesitate before situating coeliac disease within the context of the disability literature, even if the legal process treats the invocation of disability as the most viable route to public (legal) recognition of their condition.^1^ Instead, we prefer to treat coeliac disease as a unique case that offers an opportunity to look afresh at the dynamics of exclusion and inclusion in workplaces.

Beyond the medical and legal factors, coeliac disease also impacts upon workplace practices on account of social misrepresentation and stigmatisation. Caven and Nachmias (2017) illustrate one scenario where an employer referred to an employee’s coeliac diagnosis as an eating disorder in public spaces, including meetings. Transforming coeliac from a disease to an eating disorder can have serious consequences. Indeed, Veen et al. (2013, p. 592) show how impulses to demedicalise diet can also take place within families where the food consumption of the coeliac family member is treated as ‘a matter of choice rather than prescription’. Medical research shows there is a consistent lack of suitable food at work meetings and events, something which is echoed in the experiences of many people with coeliac disease: ‘… they forgot me. So everyone had cream buns except for me’ (cited in Sverker et al., 2005, p. 176).

Many coeliac‐afflicted individuals also note a loss of trust in food preparers who have proven unreliable in the claims they make about the content of their food: ‘I have been glutened too many times by the canteen’ (cited in Caven & Nachmias, 2017, p. 30). Moreover, these impacts and associated exclusions are often even more pronounced for employees who deal with ongoing symptoms: ‘… lots of trips to the bathroom, I mean it got to a ridiculous stage when I couldn’t even get to work … without having to go to the bathroom’ (cited in Crocker et al., 2018, p. 216). The tendency to demedicalise coeliac disease is consistent with tendencies found in response to other contested illnesses, carrying important consequences. This is because the downplaying of these illnesses’ physiological aspects outside of (and within) the medical profession hinders legal and social acceptance (e.g. see Phillips, 2010).

We suggest that the experience of coeliac disease within this medical, legal and social context provides an opportunity to explore how the dynamics of exclusion play out in inclusive‐orientated organisations by creating (unintended) barriers that often lead to more pronounced forms of exclusion on the grounds of misrepresentation and loss of trust. In the absence of medically approved cures, the approach to long‐term medical conditions is to emphasise personal management of the disease. This ‘individualisation’ and personal management is emphasised further for coeliac‐afflicted individuals, as not only is there no cure, but there are no prescribed medications to alleviate pain or symptoms. The impact is to individualise the illness further by emphasising diet‐management techniques and resilience imperatives. As such the workplace perceptions of coeliac disease oscillate between a private matter, concerning dietary self‐management; and a disability in its legal sense, especially when attempting to demonstrate workplace discrimination.

## THE DYNAMICS OF INCLUSION AND EXCLUSION AT WORK: MOVING BEYOND THE ‘POLITICS OF INCLUSION’ AND (EMBODIED) OTHERING

This section first surveys the way the literature on the ‘politics of inclusion’ apprehends the existing dynamics of organisational practices regarding health in the workplace, before turning to the concept of (embodied) Othering that scholars have used to characterise body‐centred exclusionary processes. Finally, we introduce the concepts of logics of difference and equivalence which we will later employ to explore the conditions which make possible the enactment of practices of exclusion. We suggest that these two logics help us characterise specific dynamics in a way that builds on insights offered by the concept of (embodied) Othering.

### The dynamics of inclusion and exclusion through the ‘politics of inclusion’ and (embodied) Othering

Considerable literature points to inequality and exclusion within workplaces (Dew & Taupo, 2009; Remnant et al., 2023) being driven—somewhat paradoxically—by a ‘politics of inclusion’ (Adamson et al., 2021, p. 213). A ‘politics of inclusion’ perspective can be said to signal a move beyond social‐managerial approaches to inclusion in a way that more decisively acknowledges the deep‐seated political character of differential workplace treatment and exclusion. For example, Adamson et al. (2021, p. 211) argue that such an approach would contribute to a ‘more nuanced understanding of inclusion by situating it in the broader social context and questioning the inclusion‐exclusion binary’. A ‘politics of inclusion’ approach accepts that realising ideals of inclusion is not reducible to a strategic game whereby people situate themselves on the side of the included or on the side of the excluded. Rather, one needs to politicise the norms that produce the inclusion/exclusion divide in the first place.

However, a ‘politics of inclusion’ approach is less clear on the process by which differential treatment is enacted in practice. To make the ‘doings’ of inclusion and exclusion visible in workplaces and to analyse their effects, a growing literature has appealed to the idea of (embodied) Othering (Dale & Latham, 2015; Harding et al., 2022; Mik‐Meyer, 2016; Remnant et al., 2023; van Amsterdam et al., 2023). Dale and Latham (2015, p. 179) suggest that:

… we need to explore how organisational processes are involved in the ‘cuts’ that form (both material and social) boundaries and differences, and produce inclusions and exclusions, inequalities and hierarchies, subjects and objects.

(Dale & Latham)

These boundaries materialise in the consequences that follow when the employee’s body fails to adhere to the employer’s participation and performance imperatives (Harding et al., 2022). Remnant et al. (2023), for example, illustrate challenges in attempting to normalise gynaecological health conditions in workplaces from the perspective of women employees. While Brewis et al. (2017) identify absence policies designed to ensure that menopausal women are not unfairly judged in taking additional sick leave to manage symptoms, Jack et al. (2019) demonstrate that these opportunities are either not taken up in practice or positively contribute to undermining the position of women in the organisation. This undermining can be caused, for example, by the worry of being ‘outed’ (othered) as menopausal when making use of dedicated organisational policies (Jack et al., 2019).

Thus, material and social embodiment in workplaces (Harding et al., 2022) tends to produce differences accompanied by effects of (embodied) Othering (Dale & Latham, 2015; Harding et al., 2022). For example, a difference often reified in organisations comprises a masculine able‐body norm, co‐constructing ‘an’ Other to which is attributed ‘an’ expectation of normalcy (Foster & Wass, 2013). We describe this difference as ‘an’ Other to foreground a tendency to dichotomise or ‘binarise’ subject positions: the construction of a hegemonic norm entails the construction of an Other that helps constitute the norm. Consequently, efforts to include a new difference as ‘normal’ tends to produce an excluded Other treated as foreign or subordinate (Mik‐Meyer, 2016).

The politics of inclusion literature, including Othering literature, offers insights about how differences are enacted in practice and how such differences are rooted not merely in strategic‐instrumental considerations but also in wider structural and political considerations. We build on these insights by paying attention to the way differences and dualisms are discursively constructed and reinforced in the workplace, with reference to people with coeliac disease. Coeliac disease‐based exclusions challenge aspects of existing frameworks because differences, though they reside in the body, are not easily ‘read off’ the body nor widely recognised, making a focus on workplace discourses about people with coeliac disease a potentially revealing area of study. We turn now to the logics of difference and equivalence, drawn from political discourse theory, as we find them helpful in elucidating the dynamics of inclusion and exclusion in a way that also points beyond a dualist horizon.

### The dynamics of inclusion and exclusion through the logics of difference and equivalence

Laclau and Mouffe’s political discourse theory assumes that the norms governing practices, including those enacted in the name of a ‘politics of inclusion’ while historically shaped, are not necessary. This contingent character makes them vulnerable to political contestation and displacement. Laclau and Mouffe’s theory comprises a range of concepts that help elucidate processes of formation, transformation and maintenance of social practices and their norms (Laclau & Mouffe, 2001).

We focus only on what they call logics of equivalence and difference to explore the dynamics of inclusion and exclusion that affect people with coeliac disease in the workplace. These concepts are analytic devices that can be used to study social and political practices discursively and which have already been applied to explore health policy discourse (Speed & Mannion, 2020).

Deploying the logics for explanatory purposes entails showing how two discursive elements can be rendered equivalent to, or different from, each other with reference to a word or expression (a ‘signifier’). For example, someone with coeliac disease may be discursively construed as similar or different to another person with reference to the signifiers ‘disability’ or ‘dietary lifestyle choice’. Suggesting that coeliac disease is *not* a ‘disability’ because it is not explicitly recognised in law is an example of the logic of difference in operation, while suggesting that coeliac disease *does* reflect a ‘dietary lifestyle choice’ would be an example of the logic of equivalence. Analysing practices as a function of the logics of equivalence and difference amounts to treating discourse as a political struggle in which different actors seek to articulate the ‘coeliac condition’ with (or dis‐articulate it from) sets of key terms, such as ‘long term medical condition’, ‘eating disorder’, ‘disability’, ‘lifestyle dietary choice’ or even ‘disease’. In proposing to study how coeliac disease is lived in the workplace, we follow scholars who have also deployed these logics to show how differences and similarities coalesce in practices, often carrying with them important material implications (Glynos & Howarth, 2007; Gunnarsson Payne & Korolczuk, 2016; Speed & Mannion, 2020).

The logics of difference and equivalence are especially useful to deploy when faced with discursive elements or differences that cannot be subsumed within existing dichotomies, such as fit‐unfit or well‐unwell. This is because both logics are explicitly tied to the creative efforts of a subject (e.g. an employee, policymaker, legislator, union representative and employment tribunal judge) to articulate new connections by mobilising signifying resources to hand. The way coeliac disease is lived in the workplace points to the need to move beyond existing dichotomic templates and visible differences not only because the autoimmune disease is predominantly invisible but also because the disease does not conform to stable dualist patterns of thought, as the employment tribunal cases show.

Many employees with coeliac disease do not themselves know what support they should expect from their workplaces (Caven & Nachmias, 2017). Thus, it is unsurprising that there is limited organisational literature available on coeliac disease (Caven & Nachmias, 2017) and that workplace challenges have predominantly been highlighted by medical scholars when exploring a patient’s life quality after a diagnosis (Crocker et al., 2018). This situation makes coeliac disease an appropriate case to study using a political discourse theory framework, as the Othering of coeliac‐afflicted individuals occurs on an ever‐shifting terrain, making the flexibility of the logics of equivalence and difference attractive from an analytical perspective.

Another feature associated with a turn to political discourse theory concerns the family resemblance the logics of equivalence and difference share with standard forms of legal reasoning as analogical. Although employment tribunal cases are not considered court cases as such, they nevertheless adopt similar forms and tropes of reasoning, which amount to establishing similarities and differences across cases, as precedents. We thus suggest that the logics are helpful concepts with which to elucidate the discursive mechanisms underlying the dynamics of exclusion and inclusion in the workplace, as they affect people with coeliac disease. Before we present our analysis of these dynamics, however, we sketch out our research methodology and its underlying rationale.

## METHODOLOGY

In this article, we explore the dynamics of exclusion as they relate to employees with coeliac disease and we do so through a critical reading of UK’s employment tribunal decisions, conceiving these as spaces of public contestation in which workplace practices are brought to light. In this section, we describe the background and procedures associated with employment tribunals in the UK, before presenting the rationale underpinning our methods of data collection and analysis.

Within the employment context, there is a first‐tier tribunal, the ‘employment tribunal’ and an upper tribunal, the ‘employment appeals tribunal’. All tribunal hearings are chaired by an ‘employment judge’, who is assisted by two lay experts in complex cases, one with experience from the perspective of an employer (such as a human resources specialist) and one who has experience from the perspective of an employee (such as a trade union official). From a procedural point of view, the parties must exhaust all internal resolution processes before filing with the UK employment tribunal, after which employees and employers are invited to make their case before the panel.

With respect to coeliac disease, complainants before the tribunal seek to demonstrate discrimination and this requires the tribunal to determine whether coeliac disease constitutes an instance of disability as envisioned by the EA 2010 and associated texts. Since there is ambiguity within the EA 2010, some tribunal decisions determine that coeliac disease is: (a) a disability and (b) a legally recognised protected difference. Other tribunal decisions find that it is not a disability or that the complainant was not suffering unduly from its effects. This decision moment constitutes a terrain of contestation, as there is no a priori reasoning to support the contention that the general rule (disability) subsumes the particular instance (coeliac disease). Instead, the tribunal panel confronts an undecidable moment by using the evidence that is brought forward in each case (Derrida, 1992).

**FIGURE 1:**
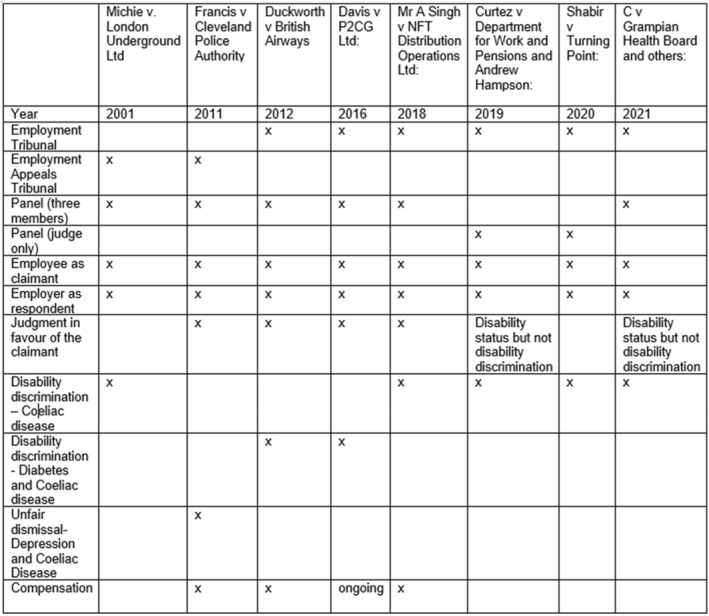
Descriptive characteristics of employment tribunals considering coeliac disease.

In constructing our corpus, we used the search terms ‘coeliac(s)’ and ‘coeliac disease’ to identify relevant cases from a database comprising all tribunal decisions filed on the UK government website and from the British and Irish Legal Information Institute. The search of 95,197 decisions yielded 11 tribunal decisions with the first record on coeliac disease in the UK appearing in 2001. From these 11, we removed three from analysis because two tribunals established that the claimants did not suffer from coeliac disease after conducting medical tests and one established that the claimant had not disclosed the disease to their employer. Each of the remaining eight tribunal decision documents summarises the key dispute, presenting the evidence, context, facts, relevant law, tribunal judgment and conclusion. The documents (reviewed judgments, judgments with reasons and where relevant, remedies), which vary between 11 and 144 pages depending on the complexity of the case, can also include excerpts of witness statements, third‐party reports and internal reports submitted by the parties involved. Figure 1 maps out relevant characteristics.

To conduct our analysis, we employed the legal method of case law analysis and discourse analysis. Case law analysis takes analogical reasoning to be central and involves the study of similarities and differences in facts and judgments across cases, which form the basis of the doctrine of the common law approach to precedent (Johnstone, 2016). To study the similarities and differences, we moved iteratively through the records of the eight tribunal judgments, reading and re‐reading the documents and noting down outcomes and the tribunals’ overall reasoning. We recorded whether the tribunals dismissed or agreed that coeliac disease was a disability, as well as the expressions used in the tribunals’ reasoning and in the witness statements and reports submitted as evidence to the panels. We focused our analysis principally on how the positions of the complainant, the respondent and the panel’s judgment were presented in the tribunal. Our analysis yielded three overarching themes of organisational practice: non‐recognition (coeliac disease being denied status as a disability or medical condition), stigmatisation (coeliac disease dismissed as easy to manage or at most a ‘special diet’) and a community theme (collective efforts to understand coeliac disease as a disability or a medical condition and to make relevant demands).

We then conducted a more detailed discourse analysis. This involved re‐reading theme‐based extracts, probing each theme using the logics of difference and equivalence with the aim of identifying mechanisms that make sense of the practices of inclusion and exclusion in the workplace. For example, the dynamics associated with the theme of non‐recognition were underpinned by a mechanism of *public omission* (on the side of the employer and statutory authorities), accompanied by a mechanism of *constrained individualisation* (on the side of the employee). Repeating this logics‐based discourse analysis for each of the other themes enabled us to identify three paired mechanisms with which to account for the workplace dynamics of exclusion in relation to employees with coeliac disease.

## ANALYSIS

### The battle for recognition: Mechanisms of public omission and constrained individualisation

Inclusion begins with public recognition of the condition, which is harder given the absence of legal stipulations or guidelines with which to establish employer obligations towards employees with coeliac disease. This vagueness is a potential source of (embodied) Othering or exclusion grounded in non‐recognition. The impact of not recognising the disease through formal organisational responses or legal channels sidelines the lived experience of coeliac‐afflicted individuals, leaving their treatment to the discretion of the organisation and individualising their struggle for inclusion. We suggest that non‐recognition exacerbates their invisibility in the workplace and generates uncertainty and anxiety for them. This non‐recognition can be captured through two mechanisms: first, an employer‐sided mechanism of *public‐official omission* and second, an employee‐sided mechanism of *constrained‐individualisation*.

The mechanism of *public official omission* manifests itself through the logics of difference and equivalence: a difference between coeliac disease and disability is established and reinforced, while an equivalence is constructed between coeliac disease and lifestyle choice. While logics of equivalence and difference are simultaneously operative in a range of fora, the former is more evident in UK legislation and official guidelines, while the latter is most evident in the tribunal cases. In establishing a difference between coeliac disease and disability in the UK law, the debate around legal discrimination and recognition depends on one’s capacity to categorise a condition as a disability. The EA 2010 specifies that ‘[c]ancer, HIV infection and multiple sclerosis are each a disability’ and as such are generally protected medical conditions under UK law (Schedule 1 EA, 2010). Intriguingly, government guidance published by the Office for Disability Issues (ODI) explicitly states that the EA 2010 refers to medical conditions including ‘auto‐immune conditions’ (2011, p. 8), but coeliac disease is not included in a list of example conditions.

The Department of Health and Social Care (DHSC) reinforces the logic of difference as it states that ‘coeliac disease is *not* defined as a disability under the EA 2010’ (2018, p. 14; emphasis added). And yet, the DHSC also presents ambiguity. For example, the DHSC does maintain that coeliac disease, albeit not a disability, is a ‘long‐term condition’. Similarly, the ODI, for example, in using diabetes as an illustration of how to treat long‐term conditions, states that the adverse effects of diabetes should be evaluated also when the person is ‘not taking (…) medication or following the required diet’ (ODI, 2011, p. 21). Thus, there is confusion as to why coeliac disease, as an autoimmune disease, should be excluded from the status of a protected difference in comparison to similar autoimmune diseases that are ‘controlled by medication or diet’ (ODI, 2011, p. 21).

The logic of difference outlined above is accompanied—as part of an overarching *mechanism of public‐official omission*—by an equally prominent logic of equivalence evident in tribunal case law. Here, coeliac disease is constructed as ‘just’ another (dietary) lifestyle choice, which over‐simplifies its complexity and thus pre‐empts the construction of a competing equivalence with a disability or any long‐term condition with debilitating symptoms that would transform it into a recognised protected difference. We see evidence of this in *Shabir v Turning Point* (2021). Shabir expected Turning Point to provide flexibility concerning shift patterns after Shabir disclosed a coeliac disease diagnosis. Shabir sought a shift pattern that would enable the preparation of gluten‐free meals at home for consumption at work. Managers at Turning Point refused this request, so Shabir brought a claim to the employment tribunal. The UK EA 2010 required that Shabir convince the tribunal that his condition constituted a disability and then to demonstrate that the actions of Turning Point amounted to discrimination. Shabir’s claim failed because the tribunal determined that Shabir’s coeliac disease was a ‘special diet[ary]’ choice, with the implication that making home‐prepared food was not necessary for the discharge of work‐related objectives (*Shabir v Turning Point*, 2021).

By pairing the logics of difference (coeliac disease is not a disability) and equivalence (coeliac disease is a lifestyle choice), we thus see how exclusionary norms in organisations can remain uncontested with respect to coeliac disease even as wider legal and organisational frameworks appear to promote ideals of inclusion. Together these logics constitute a ‘top‐down’ mechanism of *public‐official omission*: they work in such a way as to marginalise the experience of people with coeliac disease at work. But this top‐down mechanism is often accompanied by another ‘bottom‐up’ mechanism of *constrained individualisation*. This mechanism characterises the way people with coeliac disease are encouraged to internalise aspects of the mechanism of public‐official omission. In *Shabir v Turning Point* (2021, p. 5), the tribunal effectively stipulates how coeliac‐afflicted individuals should navigate their food choices, finding that ‘[g]luten‐free food … does not need to be cooked or is already prepared … and could readily be obtained …’. By emphasising this choice‐based paradigm, people with coeliac disease are encouraged to treat the challenges they face at work as an individual issue.

Yet, while Shabir initially identified with this ‘constrained individualising’ solution, its limitations soon became evident. As the food that was available to staff members at Turning Point during breaks contained gluten, Shabir turned to alternative solutions to allow himself to consume food during breaks with colleagues. In particular, Shabir asked for—and was refused—suitable takeaway food. Consequently, Shabir had to take out extra time each day to be able to consume suitable food during breaks, while colleagues took advantage of easy access to food. It was at this point that Shabir asked for a change in shift patterns to allow for the preparation of suitable meals at home to bring to work. In siding with the management’s decision to refuse this request, the tribunal stated that Shabir could not reasonably expect the employer to supply gluten‐free food because the food available to staff comprised leftovers from residents in the charity’s residential care unit (*Shabir v Turning Point*, 2021).

We see from the above how discursive logics of difference and equivalence were deployed, with the tribunal siding with the employer in rejecting Shabir’s claim that Turning Point’s decision not to vary Shabir’s shift pattern was discriminatory. What is instructive about this tribunal decision is how it draws on dominant cultural representations of coeliac disease as a lifestyle choice, ignoring medical evidence that portrays it as a complex condition that demands one pay attention to the unique experiences of people with coeliac disease. While the tribunal’s line of argument may be inclined to reinforce a logic of difference that disqualifies coeliac disease as a disability, it also reinforces a cultural logic of difference that disqualifies coeliac disease as a complex medical condition, rendering it equivalent to a lifestyle choice instead.

However inevitable these organisational practices appear to be, there are always moments of contingency that point to other potential outcomes. This can be illustrated by noting how the *Shabir v Turning Point* (2021) decision appears to contradict an earlier tribunal decision in *Curtez v Department for Work and Pension* (2020) regarding the use of medical expertise. In following medical evidence, the tribunal in *Curtez* determined that the Department for Work and Pensions should suspend late shifts for a certain period for an employee with suspected coeliac disease as it ‘interfere[d] with food intake and [a] treatment regime and may exacerbate symptoms further’ (*Curtez v Department for Work and Pension*, 2020, p. 3). We see further evidence of medical recognition in *C v Grampian Health Board and others* (2022), as the tribunal accepted that—although the employer had willingly accommodated requests to change shifts—symptoms can persist despite these adjustments and despite the employee’s condition improving ‘to an extent at least’ (*C v Grampian Health Board and others*, 2022, p. 89).

### Coeliac‐afflicted individuals as authors of their own misfortunes: The mechanisms of patronising stigmatisation and pre‐emptive withdrawal

Expecting coeliac‐afflicted individuals to find individualised solutions constructs an environment where it is likely for us to witness stigmatisation and patronising moralism that characterise practices of (embodied) Othering and exclusion. We can capture these through what we call the mechanisms of *patronising stigmatisation* and *pre‐emptive withdrawal*. While the top‐down mechanism of patronising stigmatisation has its locus in the employer, the bottom‐up mechanism of pre‐emptive withdrawal has its locus in the employee.

One example of employer‐sided *patronising stigmatisation* is evident in *Davis v P2CG Limited* (2022). Davis gave testimony that he was ridiculed by the statutory director of P2CG when disclosing a potential coeliac diagnosis on top of his diabetes. According to Davis, the statutory director exclaimed: ‘what’s happening to you man, you’re breaking down—think it’s time that we took you out to the woods and put you out of your misery, while miming shooting’ (*Davis v P2CG Limited*, 2022, p. 18). The impact of these words and acts can prompt exclusion through a logic of difference that ‘others’ people with coeliac disease as unfit for work.

Given a context primed for stigmatising reactions to people with coeliac disease, it is perhaps not surprising why some employees engage in pre‐emptive acts of *self*‐exclusion. We capture this dynamic through the mechanism of *pre‐emptive withdrawal*, which represents an employee‐sided internalised acceptance of exclusionary practices in response to actual or anticipated acts of stigmatisation. The tribunal cases offer intriguing evidence of how this mechanism plays out through the performance of the logic of difference. Here, we find instances in which the employee resists making coeliac disease equivalent to a medical condition that might otherwise attract legal recognition as a protected difference. For example, we see this in *Singh v NFT Distribution Operation Ltd* (2018). After Singh was denied a change of roles by the Depot Manager that would help manage frequent bathroom breaks, Singh did not follow up with any alternative proposals. Expecting Singh to do so could be seen as very much in sync with the mechanism of *constrained individualism*.

We emphasise here not a person’s perceived need to come up with individualised solutions, but rather their effort to *prevent* the perception of problems emerging in the first place. Thus, in the following weeks and months, Singh actively refused a referral to Occupational Health (OH) despite accumulating months of sickness absences, exemplifying the *mechanism of pre‐emptive withdrawal*.^2^ The further request for a change in duties that led to the tribunal case only came 12 months after the initial discussion with the Depot Manager (*Singh v NFT Distribution Operation Ltd*, 2018). Similarly, in the case of *C v Grampian Health Board and others* (2022) the tribunal noted how C actively minimised the transmission of health information to managers and OH, making it difficult for the employer to determine what it meant for them to be ‘OK’ while living with ‘a medical condition’ (*C v Grampian Health Board and others*, 2022, p. 79).

This mechanism of pre‐emptive withdrawal can help reinforce stigmatising behaviour. But it can also work in concert with *patronising* employer behaviour. This is especially so in relation to how some organisations often assume to know what is best for the employee’s welfare, including cases in which employees have disclosed their coeliac condition. *Duckworth v British Airways* (2012) provides such an example. Duckworth, a cabin crew member for British Airways, disclosed a coeliac diagnosis and requested a transfer from long‐haul to short‐haul flights following Duckworth’s hospitalisation after eating a gluten‐contaminated in‐flight meal. British Airways at first declined the request, then delayed taking a decision and then chose not to treat the request as a necessary work adjustment. British Airways managers used an OH‐led ‘rehabilitation plan’ for Duckworth to plan the return to long‐haul flights and argued that a transfer would have a ‘detrimental effect’ on flight attendants already serving short‐haul flights (*Duckworth v British Airways*, 2012, p. 5).

This practice, solely informed by OH advice, reinforces the sense that organisations know what is best for the employee’s body and their associated medical conditions. The British Airways decision mobilised a logic of difference within the mechanism of *patronising stigmatisation*. British Airways did not consult medical‐scientific evidence, effectively prioritising managerial shift plans and organisational schedules. Thus, the mechanism enables us to see how a patronising dimension of the mechanism operates to treat coeliac disease as different from other diseases while also demoting its status so that it is unworthy of the attention the claimant sought. Organisational norms that govern shift patterns and access to facilities, such as toilets, remain uncontested.

Finally, we note the wider sociocultural motifs that—through the operation of the logics of difference and equivalence—reinforce the exclusionary effects of the mechanisms of *patronising stigmatisation* and *pre‐emptive withdrawal*. This includes, for example, the establishment of an equivalence between coeliac disease and ‘eating disorder’, ‘lifestyle choice’, or a condition manageable through diet. Consider again the case of *Singh v NFT Distribution Operation Ltd* (2018). Following the accumulation of 135 h of sick leave over a short period, Singh’s line manager, armed with an Internet search on managing coeliac disease, questioned whether Singh ‘was strictly adhering to his diet’ (*Singh v NFT Distribution Operation Ltd*, 2018, p. 5).

The line manager, in following their Internet source, believed that coeliac symptoms would disappear with strict adherence to a gluten‐free diet and questioned the veracity of the sick notes presented for sick leave. Singh’s line manager concluded that Singh was the ‘author of his own misfortunes in being unable to perform’ at work (*Singh v NFT Distribution Operation Ltd*, 2018, p. 5). The line manager thus performs a logic of difference, privileging their understanding of coeliac disease, garnered through an Internet search, in place of Singh's lived experience. We can speculate that imperatives to maximise labour input and profit may play a role here, but these logics of difference and equivalence help construct and reinforce a mechanism of *patronising stigmatisation* that often works in concert with a mechanism of *pre‐emptive withdrawal*.

### Coeliac disease as a community integration project: Mechanisms of anti‐discrimination and democratic voice

Organisations with a supportive work environment can ‘do’ inclusion by allowing employees with coeliac disease a sense of belonging or acceptance. We can see some tribunal‐based evidence of how this practice of inclusion can be enacted through a process of community integration, comprising two mechanisms: an employer‐sided mechanism of *anti‐discrimination* and an employee‐sided mechanism of *democratic voice*. Both mechanisms are mobilised through logics of equivalence that reinforce coeliac disease’s status as a disability and medical condition and through logics of difference that marginalise competing candidates.

In *Singh v NFT Distribution Operation Ltd* (2018), *Francis v Cleveland Police Authority* (2011), *Duckworth v British Airways Plc* (2012) and *C v Grampian Health Board and others* (2022), the tribunals recognised the medical burden of coeliac disease for the employees. This dynamic of the recognition of coeliac disease as a medical burden is captured through the mechanism of *anti‐discrimination*. The upshot of both *Singh* and *Duckworth’s* tribunal decisions was that employers engaged in unlawful disability discrimination, while the *Francis* Appeal Tribunal upheld an earlier decision that the employer had engaged in unfair dismissal. These decisions demonstrate the mobilisation of the logic of equivalence insofar as the meaning of coeliac disease was rendered equivalent to that of a medical condition and, through that, a legally defined disability, while simultaneously mobilising the logic of difference to differentiate it from, and thus marginalise, competing meanings. For example, the Appeals Tribunal of *Francis v Cleveland Police Authority* (2011) argued in its judgment that tribunal decisions should be established on medical evidence and not on social assumptions as played out in the original tribunal hearing.

Moreover, in the case of *Francis*, the tribunal acknowledged that coeliac disease was ‘not responsive to diet’ (*Francis v Cleveland Police Authority*, 2011, section 14) and in *Singh v NFT Distribution Operation Ltd* (2018) it was determined, for the first time in a tribunal context, that withholding the sick pay of an employee with coeliac disease breached the UK’s EAs 2010 and constituted a disability‐based discriminatory practice. In fact, this latter judgment determined that it was inadequate to compensate the claimant for lost statutory sick pay because limiting compensation to lost statutory pay prioritised profit maximisation imperatives. Instead, the tribunal ruled that the organisation had a duty to furnish the employee with enhanced sickness support.

*Duckworth v British Airways Plc* (2012) is also interesting for another reason. Although *Duckworth* ruled in favour of the claimant on grounds of disability, the designation of disability was linked to diabetes, not coeliac disease. The tribunal held instead that coeliac disease negatively impacted *Duckworth*’s diabetes. Taken together, however, *Singh* and *Duckworth* demonstrate the opportunity for extending the scope of the category *disability* in the EA 2010 to include coeliac disease and the role discourse and the logics of equivalence and difference play in realising this opportunity. This potential expansion in the concept of disability reflects a shift away from the traditional restrictive approach to disability that focused on a set of terms regarded as its equivalent, such as ‘impairment’, ‘physical coordination’, ‘mobility’ and ‘continence’ (*Michie v London Underground Ltd*, 2001).

What we see is how subsequent cases have opened up the concept of disability. A logic of difference that emphasised ‘substantial adverse effects’ on ‘normal daily activities’ (as provided in section 6, UK EA 2010) rather than ‘impairment’, expanded the range of impacts that could qualify as disabling, including impacts on diet or going to the bathroom. In *C v Grampian Health Board and others* (2022), for example, the tribunal noted explicitly that coeliac disease ‘can be a disease with serious consequences’ and that ‘it did have sufficient consequences for the claimant that she was a disabled person under the Act’ (*C v Grampian Health Board and others*, 2022, p. 78). For this reason, such cases contain elements that can be understood to be components of a mechanism of *anti‐discrimination*.

We also discern in inchoate form an attempt to carve out a space for a complementary mechanism, which we call the mechanism of *democratic voice*. In *C v Grampian Health Board and others* (2022), for example, the tribunal noted that C could have provided her employer with more details of her medical background to better understand the extent of her disease and support the determination of her condition as a disability or other form of protected difference.

In suggesting that the claimant should supply further information to relevant authorities, the tribunal opened up a space within which employees could be encouraged to take a more active role in giving voice to their grievances. Although the tribunal had the employer or OH in mind when discussing this, the idea of ‘relevant authorities’ can be extended to also include employee unions or other forms of employee fora within which grievances can be expressed and formulated as demands that contest these and other norms of workplace practice, thus giving form to the mechanism of *democratic voice*. But fleshing out such a mechanism in more detail requires more research beyond tribunal judgments concerning coeliac disease, particularly as concerns its interaction with countervailing mechanisms that the tribunal decision may have underestimated, such as *patronising stigmatisation* and *pre‐emptive withdrawal*.

The hope is that decisions such as *Singh*, *Francis*, *Duckworth*, *C* and *Curtez* might encourage a more supportive organisational environment that recognises, respects and enhances the working experiences of employees living with coeliac disease. This might be a step towards inclusion on terms deemed appropriate by coeliac‐afflicted individuals. We note, though, that it will remain counterproductive for UK employees with coeliac disease to have to continue to prove ‘disability’ discrimination in an employment tribunal context. This is because the designation of coeliac disease as a disability is not necessarily a desirable way to gain protected difference status. Rose and Howard (2014), for example, point out how some coeliac‐afflicted individuals are uncomfortable with coeliac being associated with disease or ill health. Such disagreements stem from the differing presentations of employees with coeliac disease and highlight the central role that our three paired mechanisms play in producing distinct dynamics of inclusion and exclusion.

Coeliac disease is not homogenous and there is no one‐size‐fits‐all solution for organisations. What is concerning in our study is how, despite official pronouncements that espouse generally inclusive ideals, when it comes to people with coeliac disease, there are statutory authorities and organisations that ignore or trivialise the lived experiences of employees. While heterogeneity poses challenges for organisations, this is common for most disabilities and illnesses, so trusting and learning from employees through various mechanisms of *democratic voice* would be a good starting point for meaningful inclusion. At this stage, we have illustrated that coeliac‐afflicted individuals are still struggling to have their condition recognised by organisations, characterising associated processes as a function of three paired mechanisms supported by the performance of logics of difference and equivalence.

## DISCUSSION AND CONCLUSION

We used the logics of equivalence and difference to demonstrate how employees with coeliac disease can be othered in the workplace. To understand the underlying workplace practices and norms, we identified mechanisms of *public omission* and *constrained individualisation*, mechanisms of *patronising stigmatisation* and *pre‐emptive withdrawal* as well as mechanisms of *anti‐discrimination* and *democratic voice*. These three paired mechanisms unpack distinct processes of exclusion and inclusion. We developed this argument with reference to employment tribunal decisions, conceived as a space in which the public contestation of organisational practices toward health takes place. We recognise that, as spaces of public contestation, tribunals constitute only one possible forum among others, in which the challenges faced by employees are represented. Nonetheless, the discussions and decisions that take place in this forum offer a particularly intriguing entry point for the study of relevant organisational practices.

By examining discriminatory workplace practices targeting those afflicted with coeliac disease, we showed how contemporary workplace practices can produce troubling tendencies that are difficult to counteract (Dew & Taupo, 2009). Our analysis demonstrates how certain mechanisms amplify privatised individualising tendencies, with the effect of reinforcing exclusion. This is often a result of vague regulatory guidance that forces organisations to manage workplace health issues on their own on an ad hoc basis, often leaving relevant medical evidence out of their management decisions. This kind of environment can make it more likely that unhelpful attitudes will flourish, including stigmatising attitudes that turn people with coeliac disease into victims of their own misfortune. This further individualises the character of their struggle for recognition, leaving employees to their own devices when searching for solutions to these barriers in the work environment.

It is, however, also possible to imagine other mechanisms that can counter patronising and individualising tendencies, as we found in our analysis of the corpus, albeit in a rather suggestive and inchoate form. Such mechanisms might underpin forms of collective support in such struggles for recognition, sometimes by colleagues, line managers, or others besides. Such collective support for employees with coeliac disease is marginal in practice and for this reason, advocate organisations would be keen to promote such collective support mechanisms, for example, by persuading relevant powerholders to recognise that medical burdens should be borne collectively, not individually.

We suggest that our study does not simply introduce another instance of (embodied) Othering that can be added to the existing typologies of normal‐abnormal, fit‐unfit or well‐unwell (Ciuk et al., 2022). While such typological work is certainly valuable, the challenges that coeliac disease presents point to the need to generate new approaches and principles by which to understand in more detail not only the production of such dualisms and how their simple binary form conceal considerable complexity, but also how workplace relations might take other non‐binary forms.

We argue that invoking the logics of equivalence and difference can help elucidate these tasks, particularly as regards the complexity of the discursive operations underlying key mechanisms. Take for example, the employers in *Shabir* (2021) and *Duckworth* (2012), whose patronising attitudes of ‘knowing better’ about their employees’ dietary requirements can be understood in terms of a simultaneous mobilisation of the two logics. In critically analysing processes of exclusion and inclusion as a function of specific mechanisms built up through logics of difference and equivalence, we contribute to efforts that explore the possibility and character of post‐dualist approaches to exclusion and inclusion, suggesting there is further scope to explore the nuances and complexities of existing typologies. Beyond the existing typologies, we stress that the two logics can be applied to other contested illnesses, suggesting that the political discourse theory framework can be usefully applied to cases beyond those involving coeliac disease.

Finally, in conclusion, we suggest that, as regards efforts to promote inclusion in the workplace, there may be some merit in not only considering the widely affirmed value of pluralism but also paying more attention to the process of *pluralisation*, which seeks to register the complex dynamics underlying workplace practices (Connolly, 1995). Pluralisation goes further than pluralism because it registers the condition of possibility of pluralism and can thus promote acceptance and belonging even when subjects do not fit pre‐existing typologies. In line with Remnant et al. (2023, p. 1293), we conclude that further sociological attention needs to be paid to the places where ‘other people’s bodies are managed’ by becoming sensitive to the lived experiences and voices of affected employees.

## AUTHOR CONTRIBUTIONS

**Anne Steinhoff**: Conceptualization (equal); data curation (lead); formal analysis (lead); investigation (lead); methodology (equal); project administration (lead); writing—original draft (equal); writing—review and editing (equal). **Rebecca Warren**: Conceptualization (equal); data curation (supporting); formal analysis (supporting); investigation (supporting); methodology (equal); project administration (supporting); writing—original draft (equal); writing—review and editing (equal). **David Carter**: Conceptualization (equal); data curation (supporting); formal analysis (supporting); investigation (supporting); methodology (equal); project administration (supporting); writing—original draft (equal); writing—review and editing (equal). **Jason Glynos**: Conceptualization (equal); data curation (supporting); formal analysis (supporting); investigation (supporting); methodology (equal); project administration (supporting); writing—original draft (equal); writing—review and editing (equal).

## CONFLICT OF INTEREST STATEMENT

We certify that there is not any actual or potential conflict of interest from the researchers in writing or publishing this research article.

## ETHICS STATEMENT

Not applicable.

## PATIENT CONSENT

Not applicable.

## PERMISSION TO REPRODUCE MATERIAL FROM OTHER SOURCES

Not applicable.

## Data Availability

Data openly available in a public repository that does not issue DOIs: The data that support the findings of this study are openly available in the British and Irish Legal Information Institute (BAILII) and the HM Courts & Tribunals Service at https://www.bailii.org/ and https://www.gov.uk/employment‐tribunal‐decisions.

## ENDNOTES

^1^
While we do not explicitly engage with the sociology of disability literature, it is worth noting that many aspects of the dynamics of exclusion observed and described there resonate broadly with what we can find in the (embodied) Othering literature which we will be examining later. For example, Finkelstein’s (2001, p. 2) claim that ‘[i]t is society that disables us and disabled people are an oppressed social group’ resonates with the role attributed to organisations in Othering coeliac‐afflicted individuals, who are then forced to respond to this enforced social positioning. Both of these perspectives, in turn, differ from perspectives found elsewhere, including within the medical sociology literature, where impairment is emphasised as the central characteristic of disability. Bury (2000), for example, regards disability as an impairment that has both biological and social causes. (See Thomas (2004) for a valuable overview.)

^2^
We interpret the behaviour as pre‐emptive withdrawal based on the information available from the employment tribunal. The employment tribunal states on page six of its reasons: ‘He [Singh] indicated that he was continuing to struggle with diarrhoea. Once again, he was advised to control his diet. He said that he was doing what he could to try and keep his diet under control. He was offered the facility of an Occupational Health (OH) referral at that time but did not take up that suggestion.’ From further documentation, we see that an OH assessment was requested by managers 7 months later with no indication of OH and Singh having had any prior contact.
